# Planning Following Stroke: A Relational Complexity Approach Using the Tower of London

**DOI:** 10.3389/fnhum.2014.01032

**Published:** 2014-12-23

**Authors:** Glenda Andrews, Graeme S. Halford, Mark Chappell, Annick Maujean, David H. K. Shum

**Affiliations:** ^1^Behavioural Basis of Health Program, Griffith Health Institute, School of Applied Psychology, Griffith University, Gold Coast, QLD, Australia; ^2^Behavioural Basis of Health Program, Griffith Health Institute, School of Applied Psychology, Griffith University, Brisbane, QLD, Australia; ^3^Centre for National Research on Disability and Rehabilitation Medicine (CONROD), Griffith Health Institute, Griffith University, Meadowbrook, QLD, Australia

**Keywords:** Tower of London, planning, moves to solution, search depth, goal ambiguity, relational complexity, stroke, frontal lobes

## Abstract

Planning on the 4-disk version of the Tower of London (TOL4) was examined in stroke patients and unimpaired controls. Overall TOL4 solution scores indicated impaired planning in the frontal stroke but not non-frontal stroke patients. Consistent with the claim that processing the relations between current states, intermediate states, and goal states is a key process in planning, the domain-general relational complexity metric was a good indicator of the experienced difficulty of TOL4 problems. The relational complexity metric shared variance with task-specific metrics of moves to solution and search depth. Frontal stroke patients showed impaired planning compared to controls on problems at all three complexity levels, but at only two of the three levels of moves to solution, search depth and goal ambiguity. Non-frontal stroke patients showed impaired planning only on the most difficult quaternary-relational and high search depth problems. An independent measure of relational processing (viz., Latin square task) predicted TOL4 solution scores after controlling for stroke status and location, and executive processing (Trail Making Test). The findings suggest that planning involves a domain-general capacity for relational processing that depends on the frontal brain regions.

## Introduction

Planning is important in many areas of life and impairments in this capacity have adverse implications for independent living (Jefferson et al., 2006). Planning involves cognitive processes that depend on frontal regions of the brain (Shum et al., 2000, 2009; Unterrainer and Owen, 2006). In the current research, we examined the extent to which planning assessed using a 4-disk version of the Tower of London (TOL) is impaired in people who have suffered a stroke. A further issue relates to the nature of the cognitive processes that planning involves. More specifically, the research investigated the claim that processing the relations between current states, intermediate states, and goal states is a key process in planning (Halford et al., 1998) and that the complexity of these relations is a good indicator of the experienced difficulty of the TOL problems.

Planning in tower tasks such as the Tower of Hanoi and the TOL involves devising a sequence of moves in order to transform an initial state into a specified goal state. In the original 3-disk version of the TOL (viz., TOL3) developed by Shallice (1982), three colored disks are presented on three poles that differ in height. Respondents are required to rearrange the disks to match a target configuration (goal state) and to do so in a specified number of moves.

The results of several studies that employed the TOL3 to assess planning following traumatic brain injury (e.g., Cockburn, 1995; Rasmussen et al., 2006), suggested the need to increase the sensitivity of the TOL3 by including more difficult items. To address this issue, Tunstall (1999) developed the 4-disk version (TOL4) that includes ten items that require as many as nine moves. Shum et al. (2009) used the TOL4 to examine impairments in planning following traumatic brain injury. The patients performed more poorly than matched controls, but the impairment was specific to patients with frontal damage and to the items that required a greater number (i.e., six to nine) of moves. No planning impairment was observed on items that required fewer (i.e., two to five) moves. Planning performance in patients with no frontal damage was comparable to matched controls. The findings of Shum et al. (2009) demonstrated the importance of employing sensitive measures of planning. In that study, sensitivity was achieved by including simpler as well as more difficult problems that required fewer moves or more moves, respectively.

Moves to solution is widely used as a metric of TOL problem difficulty that has been employed in brain imaging studies and computational approaches to planning and problem solving in the TOL (e.g., Dehaene and Changeux, 1997; Newman et al., 2003). However, the number of moves to solution has been criticized as a complexity metric on the grounds that it does not sufficiently capture the cognitive processes underlying performance. Such criticisms have prompted researchers to consider alternate complexity metrics that tap different structural parameters of the tower tasks (Ward and Allport, 1997; Kaller et al., 2011, 2012; Köstering et al., 2014).

Köstering et al. (2014) examined two such factors (search depth and goal hierarchy) in the 3-disk TOL. Search depth refers to the number of intermediate moves that must be considered before the first goal move is made. When search depth is higher a longer series of intermediate moves and their interdependencies must be considered. Goal hierarchy (goal ambiguity) refers to the extent to which the correct sequential ordering of the goal moves is obvious from the specified goal state. When the goal state is vertical (i.e., all disks on the same pole), it is clear that the disk in the lowest position on the pole has to be placed before the disks in higher positions, so the sequential ordering of the moves is relatively unambiguous. When the goal state is flat (i.e., a disk on each of three poles), the sequential ordering of the moves is more ambiguous. Köstering et al. (2014) examined the effects of these two factors in a sample of normally aging adults. Adults aged from 60 to 76 years performed comparably on problems with low search depth, but performance declined significantly from 60 to 76 years on problems with high search depth. Adults over 76 years performed poorly irrespective of search depth. The effect of goal ambiguity was significant in that problems with less ambiguous goals were performed better than those with goals that were more ambiguous. However, this effect did not vary with age. The findings were interpreted as consistent with the frontal lobe theory of cognitive aging. Greater search depth imposes a higher demand on working memory, which is subserved by frontal regions, whereas increased goal ambiguity is thought to involve the striatum.

The search depth metric used by Köstering et al. (2014) to estimate the complexity of items on the 3-disk TOL is similar in some respects to the metric proposed in relational complexity theory (Halford et al., 1998). In this theory, complexity is defined in a domain-general way. It corresponds to the number of variables that are related in a cognitive representation, or the number of slots that must be filled. The simplest (unary) relations have a single slot. An example is class membership. The fact that Fido is a dog can be expressed as *dog*(Fido). Binary relations have two slots. An example is *larger-than*(elephant, mouse). Ternary relations have three slots as in *arithmetic addition*(2,3,5). Quaternary relations have four slots, as in *proportion*(2,3,6,9). More complex relations are predicted to impose higher processing loads than less complex relations. Thus, ternary relations impose a higher load than binary relations, and quaternary relations impose a higher load than ternary relations. On average, young adults can process four interacting variables in the same decision (Halford et al., 2005) consistent with a quaternary-relational limit.

The Method for Analysis of Relational Complexity (MARC) incorporates a set of principles for estimating the complexity of cognitive tasks (in terms of the metric) and the processing loads they impose (Halford et al., 2007b, 2010; Andrews and Halford, 2011). The estimates must be based on sound knowledge of how people perform the task and opportunities to reduce complexity and processing load through the use of segmentation and chunking must be taken into account. Segmentation involves decomposing (segmenting) complex tasks into less complex components that do not overload capacity and that can be processed in succession. Conceptual chunking involves recoding concepts into fewer variables. For example, the ternary-relational concept velocity, defined as *velocity* = *distance/time*, can be recoded into a unary-relational concept as when speed is indicated by the position of a pointer on a dial. However, the reduction in processing load occasioned by conceptual chunking comes at the cost of temporary loss of access to the relationships that make up the concept. For example, a unary-relational representation of velocity would not be sufficient to determine how velocity changes as a function of time or of distance, but it would be adequate if current velocity is the only variable of interest. By the principle of cognitive economy, humans will employ the least complex representation available to complete the task. More complex representations will be constructed only when less complex representations prove inadequate.

When tasks have multiple steps, task complexity corresponds to the most complex step. The processing load imposed will depend on the number of interacting variables that must be represented in parallel to perform the most complex step of the task, using the least demanding strategy available. Thus, demand corresponds to the peak load imposed during performance of the task, rather than to the total amount of processing involved. Complexity and number of steps can be manipulated independently as shown by Birney et al. (2006).

The relational complexity metric has been applied to tasks in many different content domains including transitive inference (Halford, 1984; Andrews and Halford, 1998; Andrews, 2010; Andrews and Mihelic, 2014), suppositional reasoning (Birney and Halford, 2002), categorical syllogisms (Zielinski et al., 2010), conditional reasoning (Cocchi et al., 2014), class inclusion (Halford and Leitch, 1989), inferences based on classification hierarchies (Halford et al., 2002b), card sorting (Halford et al., 2007a), balance scale reasoning (Halford et al., 2002a; Andrews et al., 2009), numerical reasoning (English and Halford, 1995; Andrews and Halford, 2002; Knox et al., 2010), and theory of mind (Andrews et al., 2003; Halford and Andrews, 2014), as well as decision making in gambling tasks (Bunch et al., 2007; Andrews et al., 2008), delay of gratification (Bunch and Andrews, 2012), reversal learning and conditional discrimination (Andrews et al., 2012), and comprehension of relative clause sentence (Andrews et al., 2006). The breadth with which the relational complexity metric has been (can be) applied contrasts with other metrics that apply to specific content domains or tasks with a specific structure.

Studies such as those cited above show that the complexity of relations that humans can process increases with age during childhood (Andrews and Halford, 2002, 2011; Bunch and Andrews, 2012), reaching quaternary relations in adulthood (Halford et al., 2005) before declining in later adulthood (Viskontas et al., 2005; Andrews and Todd, 2008).

**Figure 1:**
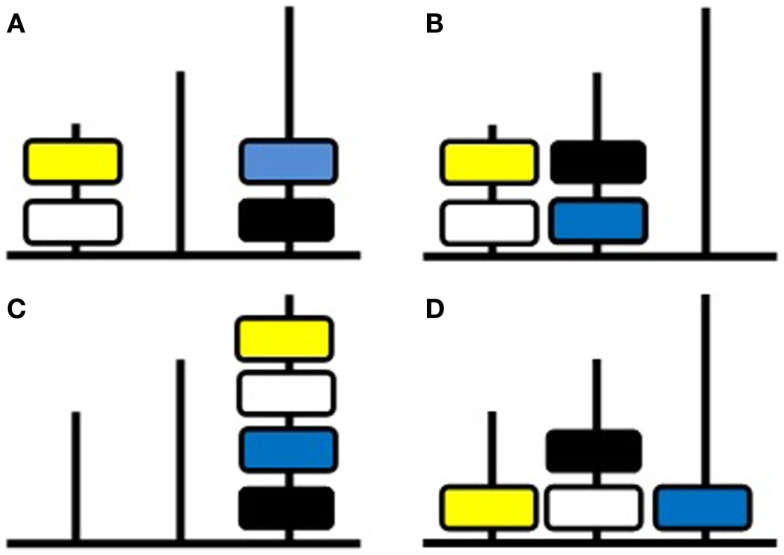
**Four-disk Tower of London (TOL4) test**. **(A)** Initial configuration used in all problems; **(B)** target configuration for the binary-relational problem described in the text; **(C)** target configuration for the ternary-relational problem described in the text; **(D)** target configuration for the quaternary-relational problem described in the text.

In the current research, we tested the hypothesis that the difficulty of TOL4 problems stems from their complexity. A relational complexity analysis of the 10 TOL4 items was conducted. The complexity analysis of three of the problems will be illustrated. The initial configuration of disks on poles was the same for all problems and it is shown in Figure 1A. The yellow (Y) and white (W) disks were on the leftmost pole (1), the blue (Bu) and black (Bk) disks were on the rightmost pole (3), while the middle pole (2) was unoccupied.

A move is coded as the binary relation, shift(color, pole). In the first problem, the goal is to transform the initial configuration (Figure 1A) into the target configuration (Figure 1B) in which yellow and white are on pole 1 and black and blue disks are on pole 2. This requires two moves. First, blue must be moved to pole 2. This is expressed as shift(Bu, 2). Second, black must be moved to pole 2. This can be expressed as shift(Bk, 2). Each move can be performed without taking any other move into account so complexity depends solely on two slots, the disk to be moved and the location to which it is moved. Therefore both moves are binary-relational, so the maximum complexity during this problem is binary-relational.

In a more complex problem, the goal is to transform the initial configuration (Figure 1A) into the target configuration (Figure 1C) in which all four disks are on pole 3 in the top-down order yellow, white, blue, and black. This problem involves nested moves. Before white can be moved to pole 3, yellow must be moved to pole 2. Nested moves such as this are coded as the higher-order relation:

prior(shift(color, pole), shift(color, pole)).

For the problem described, this sequence can be expressed as:

prior(shift(W, 3), shift(Y, 2)).

Here, there are four slots to be filled, so *prima facie* a relation between four variables is being represented. However, conceptual chunking can be employed to reduce the task to ternary-relational. In the preceding example, Y, 2 can be chunked as a single entity corresponding to “obstructing disk” (Y2) that has to be removed to enable shift(W, 3). Thus the operative variables are: disk to be shifted (W), the goal for that disk (3), and the goal for the obstructing disk (2). The principle is that the color of the obstructing disk (Y) does not need to be processed independently of the need to find a pole to shift it to, so as to remove the obstruction of shifting white to pole 3. Planning these nested moves involves ternary-relational processing. The final move involves shifting the yellow disk to pole 3, shift(Y, 3), which is binary-relational, as in the previous example. Thus the maximum complexity during this problem is ternary-relational.

In an even more complex problem, the goal is to transform the initial configuration (Figure 1A) into the target configuration (Figure 1D) in which yellow is on pole 1, black is above white on pole 2, and blue is on pole 3. This problem involves multiple nestings and conceptual chunking. Before yellow can be placed at the base of pole 1, yellow must first be moved to pole 3 so that white can be moved to pole 2. Such situations can be expressed as the higher-order relation,

prior(shift(colour, pole), prior(shift(colour, pole)), shift(colour, pole)).

These expressions can be read most easily starting at the rightmost move. Thus, in the example immediately below, Y, 3 is moved first, followed by W, 2, followed by Y, 1. For the problem described (Figure 1D), this move can be expressed as:

prior(shift(Y, 1), prior(shift(W, 2), shift(Y, 3))).

This can be chunked to quaternary-relational representation as;

prior(shift(Y, 1), prior(shift(W/Y, 2/3)))

The chunked portion can then be unpacked as;

prior(shift(W, 2), shift(Y, 3))

This yields the move to shift Y to 3 before shifting W to 2, then Y can be shifted to 1. The goal of the next move is to have blue on pole 3 and black on pole 2. To achieve this goal, blue must be first be moved to pole 1 so that black can be moved to pole 2 before blue is moved back to pole 3. This move can be expressed as,

prior(shift(Bu, 3), prior(shift(Bk, 2), shift(Bu, 1))).

As with the previous problem, chunks Bu/Bk and 2/1 can be formed, reducing the move to quaternary-relational complexity. The chunked representation can be unpacked yielding Bk on 2 and Bu on 1. Finally, Bu can be moved to 3. As in the ternary-relational problem described above, some chunking is possible. However, planning the sequence of moves will be more demanding in problems with multiple nestings because each nesting adds a new variable. By applying chunking according to the MARC principles the task can be performed with representations no more complex than quaternary-relational.

Our complexity analysis showed that the 10-item TOL4 (Shum et al., 2000, 2009) consists of two binary-relational, five ternary-relational, and three quaternary-relational problems. To ensure there were sufficient items at each complexity level, five additional items were generated, resulting in a 15-item test with three, six, and six problems at the binary-, ternary-, and quaternary-relational levels of complexity, for use in the current study.

We predicted that problems with lower estimated complexity would be easier than those with higher estimated complexity. Based on previous research demonstrating a quaternary-relational limit in young to middle adulthood (Halford et al., 2005) and age-related declines in relational processing in later adulthood (Viskontas et al., 2005; Andrews and Todd, 2008), we expected that quaternary-relational problems would be very difficult for our participants whose mean age was 66.3 years. Problem difficulty was also examined in relation to three metrics that are specific to tower tasks; namely moves to solution, goal ambiguity, and search depth.

We predicted that frontal lobe lesions would particularly impair TOL4 performance. This prediction is based on two lines of evidence. First, planning as assessed by the TOL3 has been shown to depend on the frontal regions (Newman et al., 2003; Unterrainer and Owen, 2006; Köstering et al., 2014). Second, evidence from lesion (Waltz et al., 1999, 2004; Andrews et al., 2013) and imaging studies (Kroger et al., 2002; Crone et al., 2009) has demonstrated an important role for the frontal lobes in relational processing. Therefore, if participants who have suffered a stroke affecting the frontal brain regions should show greater impairment on the TOL4 problems than those who have suffered a stroke affecting non-frontal regions or those who have not suffered a stroke, this would be consistent with the relational processing interpretation. Group differences will be examined on TOL4 problems at each level of relational complexity and at each level of moves to solution, goal ambiguity, and search depth.

A further prediction based on relational complexity theory was that an independent measure of relational processing [viz., Latin square task (LST)] would predict TOL4 solution scores after controlling for stroke status and location. This prediction was based on research demonstrating the domain-general nature of capacity to process complex relations (Halford et al., 2002a,b; Andrews et al., 2006, 2013; Birney et al., 2006, 2012; Bunch and Andrews, 2012). The predictive ability of the LST which includes items at binary, ternary, and quaternary levels of complexity was compared to the Trail Making Test (TMT), which is widely used to assess executive processes and frontal functioning. TMT was expected to account for variance in TOL4 due to the tasks’ common reliance on frontal regions (Müller et al., 2014). If the LST accounts for variance in TOL4 performance over and above the TMT this would further support the view that TOL4 involves complex relational processing.

## Materials and Methods

### Participants

The sample consisted of 83 individuals who were all native speakers of English and who were living independently in the community. Forty-three participants had brain lesions due to stroke and 40 had no known brain injury. The unimpaired individuals were recruited through sporting and social clubs. The stroke sufferers were recruited through stroke support groups in the Brisbane and Gold Coast areas in QLD, Australia. They were assigned to a frontal stroke group (*n* = 14) or a non-frontal stroke group (*n* = 29) based on neurologists’ reports and MRI/CT scan findings. Demographic details for the three groups are reported in Table 1.

**Table 1:** **Demographic details for participants in the unimpaired, non-frontal stroke, and frontal stroke groups**.

Variable		Group		
		Unimpaired	Non-frontal stroke	Frontal stroke
Age (years)	*M*	68.28	64.79	64.29
	SD	12.16	13.41	8.82
Education (years)	*M*	11.75	11.76	11.50
	SD	3.12	3.30	3.23
Gender	Males	24	19	7
	Females	16	10	7
Time since stroke (years)	*M*	–	6.05	10.08
	SE	–	0.89	1.28
MMSE	*M*	28.80	27.03	26.14
	SE	0.40	0.47	0.67

**N* = 83*.

The three groups did not differ significantly in terms of gender balance, χ^2^ (2, *N* = 83) = 0.95, *p* = 0.963, age, *F* (2, 80) = 0.94, *p* = 0.394, nor years of education, *F* (2, 80) = 0.04, *p* = 0.96. Time since stroke was significantly longer for the frontal stroke group than for the non-frontal stroke group, *t* (41) = 2.59, *p* = 0.013. To the extent that there is some recovery of function over time, this longer time since stroke would advantage the frontal stroke group over the non-frontal stroke group, thus providing a counter-confound to predicted differences between this and the other groups.

Table 2 summarizes lesion location as a function of stroke group. There was no significant association between stroke group and damage to left, right, or both hemispheres, χ^2^ (1, *N* = 43) = 3.48, *p* = 0.09, damage to temporal lobes, χ^2^ (1, *N* = 43) = 0.30, *p* = 0.73, occipital lobes, χ^2^ (1, *N* = 43) = 0.12, *p* = 0.74, sub-cortical regions, χ^2^ (1, *N* = 43) = 3.40, *p* = 0.10, nor parietal regions, χ^2^ (1, *N* = 43) = 3.85, *p* = 0.08 (exact tests).

**Table 2:** **Lesion location in the non-frontal and frontal stroke groups**.

		Stroke group	
		Non-frontal *n* = 29	Frontal *n* = 14
Hemisphere of damage	Left	11	7
	Right	16	4
	Both	2	3
Regions of damage	Temporal	8	5
	Occipital	3	1
	Sub-cortical	19	5
	Parietal	6	7
	Frontal	0	14

*Entries are frequencies*.

The Mini-Mental State Examination (MMSE; Folstein et al., 1975) was administered to all participants in the standard manner. The test consists of items assessing orientation to time and place, concentration, language, constructional ability, and immediate and delayed recall. The score was the number of correct responses (max. = 30). Mean MMSE scores are shown in Table 1. Analysis of variance (ANOVA) revealed a significant effect of group, *F* (2, 80) = 7.59, *p* = 0.001, partial η^2^ = 0.159. *Post hoc* Scheffe tests showed that the unimpaired group had significantly higher MMSE scores than the non-frontal stroke group (*p* = 0.019) and the frontal stroke group (*p* = 0.004). MMSE was therefore used as a covariate in all analyses that compared the groups.

### Measures and procedures

Ethical approval for the research was granted by the Griffith University Human Research Ethics Committee (GU Ref No: APY/82/04/HREC). Participants were tested individually at their residences by two female research assistants with postgraduate training in psychology and experience working with brain-injured individuals. The tests described below were administered as part of a larger battery. Testing was spread over two to four sessions, each 1–2 h in duration. Breaks were offered between tasks. Instructions were repeated or elaborated as required to ensure that participants understood the task requirements.

#### Tower of London

The task was an expanded 15-item version of the 4-disk TOL task of Shum et al. (2000, 2009). The apparatus consisted of four colored disks and a base with three vertical poles that differed in height and accommodated a maximum of two, three, or four disks. On all problems the apparatus was presented with the disks in the same initial configuration, which is shown in Figure 1A and Table 3. The goal states for the 15 problems are also shown in Table 3 as are the moves to solution, estimated search depth, goal ambiguity, and relational complexity for each problem.

**Table 3:** **Initial state^a^, goal states, moves to solution, relational complexity, goal ambiguity, and search depth for the 15 Tower of London problems**.

	Peg 1	Peg 2	Peg 3	Metric			
	
	Initial state		
	
	Yellow		Blue				
	White	–^b^	Black				
		
	Goal state			Moves to solution	Search depth	Goal ambiguity	Relational complexity
1	Yellow	Black		2	0	Moderate	Binary
	White	Blue	–				

2			Yellow	3	1	Low	Ternary
			White				
			Blue				
	–	–	Black				

3		Yellow	White	3	0	Moderate	Binary
	–	Blue	Black				

4		Blue		4	0	Moderate	Binary
		White					
	Black	Yellow	–				

5			Yellow	3	0	Moderate	Ternary
			White				
	–	Blue	Black				

6		Yellow		5	0	High	Ternary
	Black	Blue	White				

7	White		Yellow	5	2	Moderate	Ternary
	Blue	–	Black				

8			Blue	5	2	Low	Quaternary
			Yellow				
			White				
	–	–	Black				

9	Blue	Yellow		6	1	Moderate	Ternary
	Black	White	–				

10		Black		6	1	High	Quaternary
	Yellow	White	Blue				

11			Yellow	6	3	High	Quaternary
	White	Black	Blue				

12		Yellow		7	1	Moderate	Ternary
		Black					
	Blue	White	–				

13	Yellow			7	3	High	Quaternary
	White	Black	Blue				

14		Blue		9	5	Moderate	Quaternary
		Yellow					
	–	Black	White				

15		Blue		9	5	Moderate	Quaternary
		White					
	Yellow	Black	–				

*^a^The initial state was the same in all problems*.

*^b^Indicates an empty peg*.

Participants were instructed to rearrange the disks into the target configuration (shown pictorially), and to do so in a specified number of moves. Only one disk could be moved at a time. Scores of three, two, or one were awarded for correct solutions on the first-, second-, and third-attempts, respectively, and zero for no solution after three attempts. All participants received the problems in the order shown in Table 3 in which the problems with higher expected difficulty were concentrated later in the sequence. A stopping rule was implemented such that if participants failed to solve two consecutive problems after three attempts at each problem, no further problems were presented. The maximum score was 45 (based on 15 items). The mean number of TOL problems presented was 13.53 (SD = 2.11, range 5–15). Planning times were measured for the first attempt of each problem. Timing began at the commencement of each trial and ended when the first disk was moved. Instances of rule breaking (e.g., placing more than the allowed number of disks on a pole, moving two disks at a time) were also recorded. Rule breaks were not immediately corrected because doing so might have unduly influenced participants’ subsequent attempts on the problem.

#### Latin square task

**Figure 2:**
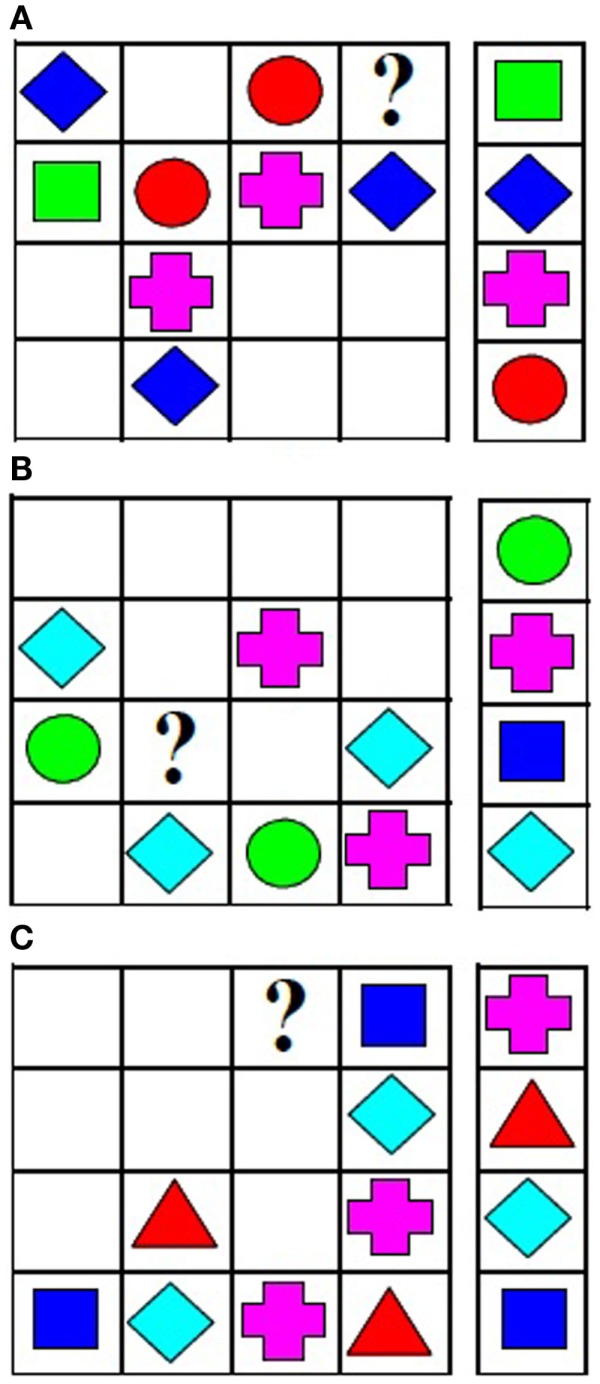
**Latin square problems at (A) binary-relational, (B) ternary-relational, and (C) quaternary-relational levels of complexity**.

On each problem on the LST task, a 4 × 4 matrix was presented on the left side of the computer screen (Birney et al., 2006, 2012; Perret et al., 2011; Andrews and Maurer, 2012). Colored geometric objects filled some cells, while other cells were empty, as shown in Figure 2. The participants’ task was to select one of four objects to fill a target cell (indicated by “?”). The response options were shown to the right of the matrix. The rule was that each of the four objects could occur only once in each row and column of the matrix. Consistent with the principles described previously, the complexity estimates reflect the most complex step within each problem.

For binary-relational problems, the most complex step required consideration of information from a single row or column. For example, the first step of the binary-relational problem shown in Figure 2A, involves working out that the empty cell in column 2 must be filled with a green square. This can be accomplished by considering the contents of a single column, column 2 in this example. On the next step, the object to be placed in the target cell can be identified by considering the contents of a single row, row 1 in this example. Row 1 now includes blue diamond, green square, and red circle, so it is clear that the pink cross must be placed in target cell. According to the analysis of Birney et al. (2006, 2012) considering the contents of a single row or a single column is binary-relational.

For ternary-relational problems, the most complex step required integration of information from a row and column. These two sources of variation must be integrated to determine the cell content. For the problem in Figure 2B, the first step is to identify the object to be placed in the cell at the intersection of column 3 and row 3 (blue square) by considering the objects already present in row 3 and column 3. Once this object is identified, the content of the target cell (pink cross) can be determined by considering the contents of row 3. The first (most complex) step is ternary-relational, whereas the second step is binary-relational.

For quaternary-relational problems, the most complex step required integration of information across multiple rows and columns. For the problem in Figure 2C, the first step is to identify the object to be placed in the cell at the intersection of column 1 and row 3 (light blue diamond) by considering the objects already present in this row and column. This step is ternary-relational. The next step requires consideration of the information in three columns (1, 2, and 4) to determine that light blue diamond should be placed in the target cell. According to the analysis provided by Birney et al. (2006, 2012) the second step is quaternary-relational.

There were four problems at each complexity level. Participants worked through the problems as quickly as possible doing all working in their heads. The score was number correct (max = 12).

#### Trail making test

In TMT Part A, numbers (1–25) were arranged randomly on a page. Participants drew lines connecting the numbers in ascending order as quickly as possible (Reitan and Wolfson, 1995). In TMT Part B, the stimuli were numbers (1–13) and letters (A–L). Participants drew lines connecting the numbers and letters in alternating order (1, A, 2, B, …). Part B required integration of two sequences (one numerical and one alphabetic) into a single alternating sequence. The two dependent measures corresponded to the times taken to complete Part A and Part B.

## Results

### Difficulty of TOL problems

Item-based correlations were computed to examine the extent of overlap among the four metrics and the extent to which each metric was associated with performance on the fifteen TOL problems. As shown in Table 4, moves to solution, search depth and relational complexity were significantly and positively inter-correlated, but the correlations with goal ambiguity did not reach significance.

**Table 4:** **Item-based correlations among moves to solution, relational complexity, goal ambiguity, search depth and solution accuracy, and planning times on the first attempt for correctly solved Tower of London problems (*N* = 15)**.

	Moves	Search depth	Goal ambiguity	Relational complexity	Solution accuracy	Planning times
**Moves to solution**						
Search depth	0.83**					
Goal ambiguity	0.28	0.05				
Relational complexity	0.75**	0.76**	0.23			
Solution accuracy	−0.90**	−0.89**	−0.32	−0.71**		
Planning times	0.81**	0.84**	0.27	0.61*	−0.90**	
Mean	5.40	1.60	1.13	3.20	2.04	22.59
*SD*	2.06	1.72	0.64	0.78	0.99	22.51

**p < 0.01; *p < 0.05.

Moves to solution, search depth and relational complexity were significantly negatively correlated with solution accuracy on the TOL problems. Solution accuracy was lower for problems that required more moves, had greater search depth and higher relational complexity. Moves to solution, search depth and relational complexity were significantly positively correlated with planning times on problems correctly solved on the first attempt. Planning times were longer for problems that required more moves, had greater search depth and higher relational complexity. Goal ambiguity was not significantly associated with solution accuracy or planning times, therefore it was not included in subsequent regression analyses.

Item-based multiple regression analyses were conducted to determine which of three metrics accounted for independent variance in solution accuracy and planning times. Given the small sample size (*N* = 15) the findings should be interpreted with caution. In the first analysis, moves to solution, search depth and relational complexity together accounted for 88% variance in solution accuracy, *F* (3, 11) = 26.85, *p* < 0.001. Moves to solution (8.29%, *p* = 0.019) and search depth (6.6%, *p* = 0.032) each accounted for unique variance. The remaining variance (73%) was shared by the predictors. In the second analysis, moves to solution, search depth, and relational complexity together accounted for 76.3% variance in planning times, *F* (3, 11) = 11.79, *p* = 0.001. Search depth accounted for unique variance (10.96%, *p* = 0.046). The remaining variance (65%) was shared by the predictors.

### TOL4 solution accuracy in stroke groups

Mini-mental state examination was included as a covariate in all analyses examining group differences. The means reported for the group based analyses have been adjusted for the covariate.

A preliminary analysis of covariance (ANCOVA) was conducted with group (unimpaired, non-frontal stroke, and frontal stroke) as the between subjects variable, and MMSE as the covariate. The dependent variable was the total score (max = 45) for the 15 TOL4 problems. The analysis yielded a significant effect of Group, *F* (2, 79) = 5.12, *p* = 0.008, partial η^2^ = 0.115. Contrast analyses showed that the difference between unimpaired group (*M* = 32.29; SE = 0.99) and the non-frontal stroke groups (*M* = 30.72; SE = 1.13) was not significant (*p* = 0.31). However, the frontal stroke group (*M* = 25.91; SE = 1.66) had significantly lower scores than the non-frontal stroke group (*p* = 0.017) and the unimpaired control group (*p* = 0.002). An analysis based on the original ten TOL4 problems yielded the same pattern of group differences.

### Sensitivity of the difficulty metrics to stroke damage

Four mixed ANCOVAs were conducted to examine group differences as a function of problem difficulty operationalized as moves, goal ambiguity, search depth, and relational complexity.

For the first analysis, the problems were categorized according to number of moves. The five low move problems required 2, 3, or 4 moves to solution, the six moderate move problems required 5 or 6 moves, and the four high move problems required 7 or 9 moves. Solution accuracy scores were converted to percentages and subjected to a mixed 3 × 3 ANCOVA in which Moves (low, moderate, and high) was a within-subject variable, Group was a between groups variable, and MMSE was the covariate. Consistent with the preceding ANCOVA and the correlations (Table 4), there were significant effects of Group, *F* (2, 79) = 4.86, *p* = 0.01, partial η^2^ = 0.11, and of Moves, *F* (2, 158) = 4.49, *p* = 0.013, partial η^2^ = 0.054. Percentage solution scores were higher for low move problems (*M* = 94.09; SE = 1.00) than for both the moderate move problems (*M* = 70.67; SE = 2.38) (*p* = 0.007) and the high move problems (*M* = 23.37; SE = 2.84) (*p* = 0.012). The Group × Moves interaction, *F* (4, 158) = 1.37, *p* = 0.25 was not significant. To facilitate comparison with other metrics, group differences were examined at each level of moves. The adjusted means are presented in Table 5.

**Table 5:** **Solution accuracy for TOL problems with low, moderate, and high moves by group**.

Group		Moves		
		Low	Moderate	High
Unimpaired	*M*	97.60	76.95	31.63
	SE	1.36	3.23	3.85
Non-frontal stroke	*M*	97.16	75.41	21.42
	SE	1.55	3.68	4.39
Frontal stroke	*M*	87.52	59.64	17.07
	SE	2.72	5.41	6.45

For low move problems, there was a significant effect of group, *F* (2, 79) = 7.78, *p* = 0.001, partial η^2^ = 0.165. Solution accuracy in the unimpaired group and non-frontal stroke did not differ significantly (*p* = 0.84). Solution accuracy in the frontal stroke group was significantly lower than the unimpaired (*p* < 0.001) and non-frontal stroke group (*p* = 0.001). For the moderate moves problems there were significant effects of the covariate, *F* (1, 79) = 4.33, *p* = 0.041, partial η^2^ = 0.052 and of group, *F* (2, 79) = 3.90, *p* = 0.024, partial η^2^ = 0.09. Solution accuracy in the unimpaired group and non-frontal stroke did not differ significantly (*p* = 0.76). Solution accuracy in the frontal stroke group was significantly lower than the unimpaired (*p* = 0.009) and non-frontal stroke group (*p* = 0.016). For the high moves problems there was no significant effect of group, *F* (2, 79) = 2.31, *p* = 0.11, partial η^2^ = 0.055.

A similar approach was used to examine goal ambiguity. There were two problems with low goal ambiguity, nine with moderate goal ambiguity, and four with high goal ambiguity. There were significant effects of Group, *F* (2, 79) = 5.40, *p* = 0.006, partial η^2^ = 0.12, and Goal Ambiguity, *F* (2, 158) = 4.32, *p* = 0.015, partial η^2^ = 0.052. Percentage solution scores were significantly higher for low goal ambiguity (*M* = 89.28; *SE* = 2.05) than high ambiguity (*M* = 53.82; *SE* = 2.97) problems, *F* (1, 79) = 6.13, *p* = 0.015, η^2^ = 0.072, and marginally higher than for problems with the moderate goal ambiguity (*M* = 66.01; SE = 1.44), *F* (1, 79) = 3.60, *p* = 0.061, η^2^ = 0.044. The Group × Goal Ambiguity interaction, *F* (4, 158) < 1, *p* = 0.55, did not approach significance. Group differences for problems with low, moderate, and high goal ambiguity were examined. The adjusted means are presented in Table 6.

**Table 6:** **Solution accuracy for TOL4 problems with low, medium, and high goal ambiguity by group**.

Group		Goal ambiguity		
		Low	Medium	High
Unimpaired	*M*	95.78	70.60	62.31
	SE	2.78	1.95	4.02
Non-frontal stroke	*M*	93.99	68.84	54.10
	SE	3.17	2.23	4.58
Frontal stroke	*M*	78.09	58.59	45.05
	SE	4.66	3.27	6.73

For problems with low goal ambiguity, there was a significant effect of group, *F* (2, 79) = 5.44, *p* = 0.006, partial η^2^ = 0.121. Solution accuracy in the unimpaired group and non-frontal stroke did not differ significantly (*p* = 0.68). Solution accuracy in the frontal stroke group was significantly lower than the unimpaired (*p* = 0.002) and non-frontal stroke group (*p* = 0.005). For problems with moderate goal ambiguity there was a significant effect of group, *F* (2, 79) = 4.92, *p* = 0.01, partial η^2^ = 0.111. Solution accuracy in the unimpaired group and non-frontal stroke did not differ significantly (*p* = 0.56). Solution accuracy in the frontal stroke group was significantly lower than the unimpaired (*p* = 0.003) and non-frontal stroke group (*p* = 0.01). For problems with high goal ambiguity there was no significant effect of group, *F* (2, 79) = 2.40, *p* = 0.097, partial η^2^ = 0.057.

Search depth was examined in the same way. The five low search depth problems had a depth of zero, the six medium depth problems had depths of 1 or 2, and the four high search depth problems had depths of 3 or 5. There were significant effects of Group, *F* (2, 79) = 5.36, *p* = 0.007, partial η^2^ = 0.12, and Search Depth, *F* (2, 158) = 4.55, *p* = 0.012, partial η^2^ = 0.054. Percentage solution scores were significantly higher for low depth (*M* = 92.55; SE = 1.14) than high depth (*M* = 16.33; SE = 2.38) problems, *F* (1, 79) = 8.30, *p* = 0.005, η^2^ = 0.095. Solution accuracy for the moderate depth (*M* = 75.74; SE = 2.43) problems did not differ significantly from low (*p* = 0.11) or high depth problems (*p* = 0.15). The Group × Search Depth interaction, *F* (4, 158) = 2.27, *p* = 0.065, partial η^2^ = 0.054, approached significance. Group differences were examined at each level of search depth. The adjusted means are shown in Table 7.

**Table 7:** **Solution accuracy for TOL4 problems with low, moderate, and high search depth by group**.

Group		Search depth		
		Low	Medium	High
Unimpaired	*M*	96.23	80.69	25.60
	SE	1.54	3.29	3.22
Non-frontal stroke	*M*	97.63	79.55	13.49
	SE	1.76	3.75	3.67
Frontal stroke	*M*	83.79	66.98	9.90
	SE	2.59	5.50	5.39

For low depth problems, there were significant effects of the covariate (MMSE), *F* (1, 79) = 4.47, *p* < 0.038, η^2^ = 0.053, and Group, *F* (2, 79) = 10.90, *p* < 0.001, η^2^ = 0.216. Solution accuracy in the unimpaired and non-frontal stroke groups did not differ significantly (*p* = 0.56). Solution accuracy in the frontal stroke group was significantly lower than in the non-frontal stroke group (*p* < 0.001) and the unimpaired group (*p* < 0.001). For the moderate depth problems, solution accuracy in the unimpaired, non-frontal, and frontal stroke groups did not differ significantly, *F* (2, 79) = 2.37, *p* = 0.10, η^2^ = 0.057. For high depth problems, there was a significant effect of Group, *F* (2, 79) = 4.19, *p* = 0.019, η^2^ = 0.096. Solution accuracy in the unimpaired group was significantly higher than in the non-frontal stroke group (*p* = 0.018) and significantly higher than in the frontal stroke group (*p* = 0.018) but the two stroke groups did not differ significantly (*p* = 0.577).

Relational complexity was examined in the same way. There were three, six, and six problems, respectively at the binary, ternary, and quaternary-relational levels of complexity. The analysis yielded significant effects of Group, *F* (2, 79) = 5.65, *p* = 0.005, partial η^2^ = 0.125 and Complexity, *F* (2, 158) = 5.23, *p* = 0.006, η^2^ = 0.062. Solution accuracy was significantly higher for binary- (*M* = 96.08; SE = 0.86) than ternary-relational problems (*M* = 78.63; SE = 2.05), *F* (1, 79) = 4.07, *p* = 0.047, η^2^ = 0.049, and for binary- than quaternary-relational problems (*M* = 37.99; SE = 2.35), *F* (1, 79) = 8.85, *p* = 0.004, η^2^ = 0.101. There was also a significant Group × Complexity interaction, *F* (4, 158) = 2.43, *p* = 0.05, η^2^ = 0.058. Group differences were examined at each complexity level. The adjusted means are shown in Table 8.

**Table 8:** **Solution accuracy for binary-, ternary-, and quaternary-relational TOL4 problems by group**.

		Relational complexity		
		Binary	Ternary	Quaternary
Unimpaired	*M*	98.18	83.55	46.73
	SE	1.16	2.78	3.18
Non-frontal stroke	*M*	99.32	83.90	37.09
	SE	1.32	3.17	3.63
Frontal stroke	*M*	90.75	68.43	30.14
	SE	1.94	4.66	5.32

For binary-relational problems, there were significant effects of the covariate (MMSE), *F* (1, 79) = 4.57, *p* < 0.036, η^2^ = 0.055, and Group, *F* (2, 79) = 7.30, *p* = 0.001, η^2^ = 0.156. Solution accuracy in the unimpaired and the non-frontal stroke groups did not differ significantly (*p* = 0.53). Solution accuracy was significantly lower in the frontal stroke group than the non-frontal stroke group (*p* < 0.001) and the unimpaired group (*p* = 0.002). For the ternary-relational problems, there was a significant effect of Group, *F* (2, 79) = 4.47, *p* = 0.015, η^2^ = 0.102. Solution accuracy in the unimpaired and the non-frontal stroke groups did not differ significantly (*p* = 0.94). Solution accuracy was significantly lower in the frontal stroke group than the non-frontal stroke group (*p* = 0.006) and the unimpaired group (*p* = 0.008). For quaternary-relational problems, there was a significant effect of Group, *F* (2, 79) = 3.85, *p* = 0.025, partial η^2^ = 0.089. Solution accuracy was marginally higher in the unimpaired group than the non-frontal stroke group (*p* = 0.054) and significantly higher than in the frontal stroke group (*p* = 0.011). The two stroke groups did not differ significantly (*p* = 0.274).

In summary, the foregoing analyses show that patterns of group differences on problems at low, intermediate, and high difficulty levels differ according to how problem difficulty is measured. On the easiest problems, the frontal stroke group performed more poorly than the unimpaired group irrespective of whether problem difficulty was expressed in terms of moves to solution, goal ambiguity, search depth, or relational complexity. The frontal stroke group also performed more poorly than the non-frontal stroke group on the easiest problems.

On problems with an intermediate level of difficulty, the frontal stroke group performed more poorly than the unimpaired group and the non-frontal stroke group when problem difficulty was expressed in terms of moves to solution, goal ambiguity, and relational complexity, but not when difficulty was expressed in terms of search depth. No significant group differences were observed on moderate depth problems.

On problems at the highest level of difficulty, the frontal stroke group performed more poorly than the unimpaired group when problem difficulty was expressed in terms of search depth and relational complexity. The frontal and non-frontal stroke groups performed poorly on high search depth and quaternary-relational problems and there were no significant differences between these two groups. No significant differences were observed between unimpaired, non-frontal stroke, and frontal stroke groups on high move problems and problems with high goal ambiguity.

Thus the pattern of significance for the group effects shows that TOL4 problems at all three levels of the domain-general relational complexity metric were sensitive to frontal lobe damage whereas TOL4 problems at two levels of the task-specific metrics (moves, goal ambiguity, and search depth) were sensitive to frontal lobe damage. Inspection of the effect sizes reported above indicates a similar pattern in that effect sizes were <0.058 for the moderate search depth, high moves, high goal ambiguity problems for which the group effect was not significant, whereas effects sizes exceeded 0.088 in all other conditions.

### Planning times for TOL problems solved on first attempt

Participants with no first attempt solutions for any problems at a particular difficulty level were excluded from these analyses. This meant that the overall sample sizes were reduced to 39 (*n* = 22 unimpaired; *n* = 12 non-frontal stroke; *n* = 5 frontal stroke) for the analysis examining moves to solution, to 72 (*n* = 35 unimpaired; *n* = 27 non-frontal stroke; *n* = 10 frontal stroke) for the analysis examining goal ambiguity, to 28 (*n* = 22 unimpaired; *n* = 4 non-frontal stroke; *n* = 2 frontal stroke) for the analysis examining search depth and to 75 (*n* = 38 unimpaired; *n* = 27 non-frontal stroke; *n* = 10 frontal stroke) for the analysis examining relational complexity. The losses were due mainly to the more difficult problems, where participants were more likely to require multiple attempts.

Four separate ANOVAs were conducted with moves, goal ambiguity, search depth, or relational complexity as the within-subject factor. There was a significant effect of Moves, *F* (2, 76) = 20.72, *p* < 0.001, partial η^2^ = 0.353. Planning times (seconds) were significantly shorter for low move problems (*M* = 8.22; SE = 0.52) than for moderate move problems (*M* = 15.27; SE = 1.56), *F* (1, 38) = 19.87, *p* < 0.001, partial η^2^ = 0.343, which were significantly shorter than for high move problems (*M* = 30.71; SE = 4.71), *F* (1, 38) = 17.17, *p* < 0.001, partial η^2^ = 0.311.

There was a significant effect of Goal Ambiguity, *F* (2, 142) = 21.36, *p* < 0.001, partial η^2^ = 0.231. Planning times (seconds) for problems with low (*M* = 10.73; SE = 1.06) and moderate goal ambiguity (*M* = 11.93; SE = 0.76) did not differ significantly, *F* (1, 71) = 1.18, *p* = 0.282. Planning tomes for problems with low ambiguity were significantly shorter than for problems with high goal ambiguity (*M* = 19.69; SE = 1.91), *F* (1, 71) = 28.897, *p* < 0.001, partial η^2^ = 0.289.

There was a significant effect of Search Depth, *F* (2, 54) = 23.48, *p* < 0.001, partial η^2^ = 0.465. Planning times were significantly shorter for low depth problems (*M* = 7.81; SE = 0.60) than for medium depth problems (*M* = 13.90; SE = 1.30), *F* (1, 27) = 22.68, *p* < 0.001, partial η^2^ = 0.467, which were significantly shorter than for high depth problems (*M* = 34.76; SE = 5.43), *F* (1, 27) = 20.87, *p* < 0.001, partial η^2^ = 0.436.

There was a significant effect of Relational Complexity, *F* (2, 148) = 14.52, *p* < 0.001, partial η^2^ = 0.164. Planning times were significantly shorter for binary-relational problems (*M* = 8.59; SE = 0.52) than for ternary-relational problems (*M* = 11.18; SE = 0.73), *F* (1, 74) = 13.80, *p* < 0.001, partial η^2^ = 0.157, which were significantly shorter than for quaternary-relational problems (*M* = 19.19; SE = 2.08), *F* (1, 74) = 11.72, *p* < 0.001, partial η^2^ = 0.137.

These findings are generally consistent with the item-based correlations. However, when Group was included as an independent variable along with MMSE as the covariate, the ANCOVAs yielded no significant effects of Group, MMSE, Moves, Goal Ambiguity, Depth or Relational Complexity, and no significant interactions. These null results likely reflect inclusion of the covariate, the small and unequal sizes of the unimpaired, non-frontal stroke and frontal stroke groups, and high within-group variability in planning times.

### TOL rule breaks

Analysis of covariance was applied to the number of rule breaks. The analysis yielded a significant effect of Group, *F* (2, 79) = 5.03, *p* = 0.009, partial η^2^ = 0.113. Contrast analyses showed that the difference between the unimpaired group (*M* = 0.35; SE = 0.21) and the non-frontal stroke group (*M* = 0.38; SE = 0.23) was not significant (*p* = 0.938). The frontal stroke group (*M* = 1.56; SE = 0.34) committed significantly more rule breaks than the non-frontal stroke group (*p* = 0.005), however it should be noted that the absolute number of rule breaks was quite low (*M* = 0.77; SE = 0.15; *N* = 83).

### Predicting TOL solution accuracy

Table 9 shows the zero-order correlations among the TOL4, LST scores (max = 12) and TMT-Parts A and B. Stroke status (0 = unimpaired; 1 = stroke) and frontal location (0 = no frontal injury; 1 = frontal injury) were dummy variables that together capture the grouping variable used in the ANCOVAs. The TOL4 measure is the average of the binary-, ternary-, and quaternary-relational percentages scores. The results are very similar when the total score (max 45) is used. The negative correlations occur because TMT-A and TMT-B are measures of response times rather than accuracy.

**Table 9:** **Zero-order correlations (*N* = 83)**.

	TOL4 (%)	Stroke status	Frontal	MMSE	TMT-A	TMT-B	LST
Stroke status	−0.31**						
Frontal location	−0.40***	0.43***					
MMSE	0.33**	−0.38***	−0.27**				
TMT-A (times)	−0.51***	0.34**	0.35**	−0.51***			
TMT-B (times)	−0.62***	0.38***	0.44***	−0.55***	0.85***		
Latin square	0.51***	−0.32**	−0.33**	0.29**	−0.42***	−0.50***	
Mean	73.00	0.52	0.17	27.74	43.94	146.01	5.78
SD	13.10	0.50	0.38	2.70	27.92	157.06	3.31

*TOL, Tower of London; TMT-A, trail making test part A completion times; TMT-B, trail making test part B completion times; LST, Latin square task*.

**p < 0.01; ***p < 0.001.

A multiple regression analysis with TOL4 as the criterion variable was conducted. On step 1, the dummy variables stroke status and frontal-non-frontal were entered, along with MMSE. These variables together accounted for significant variance in TOL4 performance. On step 2, TMT-A and TMT-B accounted for an additional 18.6% variance (*p* < 0.001). On step 3, LST accounted for a further 4.41% variance (*p* = 0.016). The unique contribution of TMT-B was reduced from 8.47% at step 2 to 5.38% at step 3, indicating that TMT-B and LST accounted for shared variance in TOL performance. This analysis is summarized in Table 10.

**Table 10:** **Multiple regression analyses predicting TOL4 solution accuracy**.

	Predictors	*B*	SE (*B*)	ß	Part	*p*
Step 1	Stroke status	−2.72	3.02	−0.10	−0.09	0.372
	Frontal/non-frontal	−10.33	3.87	−0.30	−0.27	0.009
	MMSE	1.01	0.53	0.21	0.19	0.060
	Multiple *R*^2^ = 0.218, *F* (3, 79) = 7.35, *p* < 0.001					
Step 2	Stroke status	−1.47	2.69	−0.06	−0.05	0.586
	Frontal/non-frontal	−4.90	3.60	−0.14	−0.12	0.177
	MMSE	−0.16	0.53	−0.03	−0.03	0.765
	TMT-A	0.03	0.08	0.05	0.03	0.753
	TMT-B	−0.05	0.02	−0.60	−0.29	0.001
	Multiple *R*^2^ = 0.405 *F* (5, 77) = 10.47, *p* < 0.001					
Step 3	Stroke status	−0.67	2.62	−0.03	−0.02	0.800
	Frontal/non-frontal	−4.04	3.50	−0.12	−0.10	0.252
	MMSE	−0.15	0.51	−0.03	−0.03	0.771
	TMT-A	0.02	0.08	0.05	0.03	0.770
	TMT-B	−0.04	0.02	−0.49	−0.23	0.008
	LST	0.98	0.40	0.25	0.21	0.016
	Multiple *R*^2^ = 0.449, *F* (6, 76) = 10.32, *p* < 0.001					

## Discussion

Our research examined planning assessed using a 4-disk version of the TOL (Shum et al., 2009) following stroke. The overall solution scores provided evidence of impairment but only in those whose strokes resulted in damage to frontal regions of the brain. The overall solution scores, which collapse over problem difficulty, provided no evidence of planning impairments following stroke affecting non-frontal brain regions. These findings are consistent with previous research using the TOL4 (Shum et al., 2009).

We also investigated the extent to which relational complexity theory (Halford et al., 1998), which has been shown to account for performance in many cognitive domains also applies to planning on the TOL4. According to relational complexity theory, integrating the relations between current states, intermediate states, and goal states is a key process in planning. Three aspects of the findings are consistent with relational complexity theory.

First, the observed difficulty of the TOL4 problems increased with the estimated relational complexity of the problems. This was also the case for other complexity metrics. The item-based correlations demonstrate that moves to solution, search depth, and relational complexity are not independent. In the regression analyses, search depth and moves to solution emerged as predictors of solution accuracy and search depth also predicted planning times on problems correctly solved on the first attempt, but in both cases the majority of the variance was shared. Search depth and moves to solution are intrinsic to the TOL4 task but unlike relational complexity they are not applicable across domains.

Search depth quantifies difficulty up to the first goal move. Köstering et al. (2014) showed that search depth is well suited to TOL3 problems. Our findings show that it also captures the difficulty of TOL4 problems that require up to nine moves to solution. The search depth metric and the relational complexity metric both focus on the relations and interdependencies within a sequence of moves and this might underpin the observed positive correlation.

That the number of moves metric predicted solution accuracy is consistent with many previous findings (e.g., Newman et al., 2003; Kaller et al., 2012). The finding is unsurprising in one sense because problems that require more moves to solution also provide more opportunities for errors. Nevertheless, the fact that number of moves was strongly correlated with search depth and relational complexity, which are less vulnerable to this criticism indicates its usefulness as a difficulty metric. One feature of the moves metric that might contribute to its prediction of performance is its scaling. For problems used in the current study, moves ranged from 2 to 9 with most intermediate values represented. The values of search depth (0, 1, 2, 3, 5), goal ambiguity (low, moderate, and high), and relational complexity (binary-, ternary-, and quaternary-relational) were more limited in range. These scaling differences between the metrics should be considered when interpreting the item-based correlations and regression analyses.

It is also likely that metrics that are specific to a task, as moves to solution and search depth are to TOL, will tend to account for more variance in that task. However, because such metrics cannot be applied to other tasks, they cannot be used to compare difficulty of TOL problems with other tasks. The relational complexity approach does allow this. For example, number of moves on the TOL4 task does not have the same meaning as number of moves (steps to solution) on the LST, whereas the relational complexity values are arguably comparable.

The second finding consistent with relational complexity theory is that as in previous studies (Unterrainer and Owen, 2006; Shum et al., 2009) impaired performance was most evident in people with frontal lobe damage. Relational processing is known to rely on the integrity of the frontal lobes (e.g., Waltz et al., 1999, 2004; Kroger et al., 2002; Crone et al., 2009; Andrews et al., 2013), so this finding is consistent with the view that TOL4 problems involve relational processing.

The frontal stroke group was impaired relative to unimpaired controls on TOL4 problems at all three levels of relational complexity. This was not the case when difficulty was expressed in terms of moves, goal ambiguity, and search depth. TOL4 problems with low and moderate numbers of moves, low and moderate goal ambiguity, and low and high search depth were sensitive to frontal lobe damage. Thus relational complexity was more sensitive to frontal lobe damage than the other metrics were.

Relative to the non-frontal stroke group, the frontal stroke group was impaired on low move and moderate move problems, problems with low and moderate goal ambiguity, and problems with low search depth and binary- and ternary-relational problems. Thus none of the metrics was successful in distinguishing patients with frontal versus non-frontal damage at all three levels of difficulty. The significant group effects that were observed on the most difficult quaternary-relational and high search depth problems reflected differences between unimpaired and stroke groups rather than between non-frontal and frontal stroke groups. That this impairment in the non-frontal group was detected only on a subset of the problems illustrates one benefit of analyzing the cognitive demands involved in planning on the TOL4.

Given the demonstrated limit for young adults (Halford et al., 2005), the poor performance of the two stroke groups on the quaternary-relational problems is not surprising. Recent brain imaging of individuals without brain damage showed that limits in relational processing during a deductive reasoning task were manifested in the brain as complexity-dependent modulations of large-scale networks that involved both frontal and non-frontal (e.g., parietal, occipital) regions (Cocchi et al., 2014). If these regions are damaged in individuals in the non-frontal stroke group, their performance on the quaternary-relational TOL4 problems would be adversely affected relative to the unimpaired group. Four of the six quaternary-relational problems were classified as high search depth, and this overlap would explain the similar pattern observed on the high search depth and quaternary-relational problems.

A third finding is consistent with relational complexity theory. As noted, the relational complexity approach has been applied to tasks in many different content domains and cross-domain correspondences in performance have been demonstrated in children (Andrews and Halford, 2002; Halford et al., 2002a,b; Bunch and Andrews, 2012), and adults (Andrews et al., 2006, 2013), suggesting that relational processing is a domain-general capacity. As predicted, relational processing in the LST accounted for variance in TOL4 performance after controlling for stroke status and location, MMSE and completion times on parts A and B of the TMT. The TOL4 and the LST differ substantially in terms of their stimuli and procedural requirements. Therefore the shared variance is unlikely to reflect common surface features of the tasks. We interpret the variance shared by TOL4 and LST as evidence that a common capacity for complex relational processing underpins both tasks.

Completion times for the TMT also accounted for variance in TOL4, but this was due mainly to part B rather than part A. Whereas TMT-A and TMT-B both require non-executive processes involved in visual scanning and speeded motor responses, TMT-B also requires the executive processes involved in set-shifting, maintaining two response sets in working memory, and inhibitory control (Müller et al., 2014). The unique contribution of TMT-B on step 2 of the regression analysis is consistent with the involvement of executive processes in TOL4.

As well as accounting for independent variance in TOL4, TMT-B, and LST also accounted for shared variance in TOL4. This suggests that all three tasks have some common processes. We argued previously that relational processing underpins both TOL4 and LST. TMT-B can also be construed in this way. It requires integration of two well-known sequences, one numerical and the other alphabetic. Each sequence incorporates a succession relation, in that one element is succeeded by the next element, for example, *succeeded by*(3, 4) or *succeeded by*(D, E). Succession is a binary relation because it cannot be defined on fewer than two entities. TMT-B involves integrating the numerical and alphabetic sequences such that the categories (numbers, letters) alternate, for example, *alternating*(3, D, 4). Alternation is ternary-relational because it cannot be defined on fewer than three entities. Thus we propose that the variance shared by the three tasks reflects ternary-relational processing. Some LST and TOL4 problems require quaternary-relational processing, so the unique contribution of LST might reflect this higher complexity.

The research contributes to our understanding of the processes involved in TOL4. It adds to the studies cited previously, which demonstrate that relational processing underpins performance on a wide range of cognitive tasks. Given the ubiquitous nature of relational processing, and the demonstrated effects of relational complexity on performance, relational complexity theory provides a parsimonious approach to conceptualizing human cognition.

The research also has practical implications. To the extent that planning on tower tasks can be construed as relational processing, interventions designed to improve relational processing through for example, structural alignment training (Son et al., 2011; Hribar et al., 2012), use of relational language (Gentner et al., 2011), and techniques to improve access to relational components (e.g., Andrews et al., 2012) might also have beneficial effects on planning. Thus the findings have the potential to inform cognitive rehabilitation of planning deficits following brain injury due to stroke and other factors. Impairments in planning have adverse implications for independent living (Jefferson et al., 2006). For example, without the ability to plan, a person might have problems in achieving independent activities of daily living or their vocational goals. Thus effective interventions would imply considerable benefits for individuals as well as for society more broadly.

## Conflict of Interest Statement

The authors declare that the research was conducted in the absence of any commercial or financial relationships that could be construed as a potential conflict of interest.
